# Examining the Latent Structure and Correlates of Sensory Reactivity in Autism: A Multi-site Integrative Data Analysis by the Autism Sensory Research Consortium

**DOI:** 10.21203/rs.3.rs-2447849/v1

**Published:** 2023-01-10

**Authors:** Zachary J. Williams, Roseann Schaaf, Karla K. Ausderau, Grace T. Baranek, D. Jonah Barrett, Carissa J. Cascio, Rachel L. Dumont, Ekomobong E. Eyoh, Michelle D. Failla, Jacob I. Feldman, Jennifer H. Foss-Feig, Heather L. Green, Shulamite A. Green, Jason L. He, Elizabeth A. Kaplan-Kahn, Bahar Keçeli-Kaysılı, Keren MacLennan, Zoe Mailloux, Elysa J. Marco, Lisa E. Mash, Elizabeth P. McKernan, Sophie Molholm, Stewart H. Mostofsky, Nicolaas A. J. Puts, Caroline E. Robertson, Natalie Russo, Nicole Shea, John Sideris, James S. Sutcliffe, Teresa Tavassoli, Mark T. Wallace, Ericka L. Wodka, Tiffany G. Woynaroski

**Affiliations:** Vanderbilt University School of Medicine; Thomas Jefferson University; University of Wisconsin–Madison; University of Southern California; Vanderbilt University; Vanderbilt University Medical Center; Thomas Jefferson University; University of Minnesota; The Ohio State University; Vanderbilt University Medical Center; Icahn School of Medicine at Mount Sinai; Children's Hospital of Philadelphia; University of California, Los Angeles; King's College London; Syracuse University; Vanderbilt University Medical Center; University of Reading; Thomas Jefferson University; Cortica Healthcare; Baylor College of Medicine; Syracuse University; Albert Einstein College of Medicine; Kennedy Krieger Institute; King's College London; Dartmouth College; Syracuse University; Syracuse University; University of Southern California; Vanderbilt University; University of Reading; Vanderbilt University; Kennedy Krieger Institute; Vanderbilt University Medical Center

**Keywords:** autism, integrative data analysis, meta-analysis, sensory features, sensory seeking, hyporeactivity, hyperreactivity, item response theory, sensitivity, responsiveness, measurement

## Abstract

**Background:**

Differences in responding to sensory stimuli, including sensory hyperreactivity (HYPER), hyporeactivity (HYPO), and sensory seeking (SEEK) have been observed in autistic individuals across sensory modalities, but few studies have examined the structure of these “supra-modal” traits in the autistic population.

**Methods:**

Leveraging a combined sample of 3,868 autistic youth drawn from 12 distinct data sources (ages 3–18 years and representing the full range of cognitive ability), the current study used modern psychometric and meta-analytic techniques to interrogate the latent structure and correlates of caregiver-reported HYPER, HYPO, and SEEK within and across sensory modalities. Bifactor statistical indices were used to both evaluate the strength of a “general response pattern” factor for each supra-modal construct and determine the added value of “modality-specific response pattern” scores (e.g., (Visual HYPER). Bayesian random-effects integrative data analysis models were used to examine the clinical and demographic correlates of all interpretable HYPER, HYPO and SEEK (sub)constructs.

**Results:**

All modality-specific HYPER subconstructs could be reliably and validly measured, whereas certain modality-specific HYPO and SEEK subconstructs were psychometrically inadequate when measured using existing items. Bifactor analyses unambiguously supported the validity of a supra-modal HYPER construct (ω_H_ = .800), whereas a coherent supra-modal HYPO construct was not supported (ω_H_ = .611), and supra-modal SEEK models suggested a more limited version of the construct that excluded some sensory modalities (ω_H_ = .799; 4/7 modalities). Within each sensory construct, modality-specific subscales demonstrated substantial added value beyond the supra-modal score. Meta-analytic correlations varied by construct, although sensory features tended to correlate most strongly with other domains of core autism features and co-occurring psychiatric symptoms. Certain subconstructs within the HYPO and SEEK domains were also associated with lower adaptive behavior scores.

**Limitations:**

Conclusions may not be generalizable beyond the specific pool of items used in the current study, which was limited to parent-report of observable behaviors and excluded multisensory items that reflect many “real-world” sensory experiences.

**Conclusion:**

Psychometric issues may limit the degree to which some measures of supra-modal HYPO/SEEK can be interpreted. Depending on the research question at hand, modality-specific response pattern scores may represent a valid alternative method of characterizing sensory reactivity in autism.

## Introduction

Differences in behavioral responses to sensory inputs from the environment have been associated with autism spectrum disorder (hereafter “autism”) since the first clinical descriptions of the condition (6, 7). Sensory phenotypes are present across multiple modalities (e.g., (auditory, visual, tactile) and include differences in sensory reactivity and modulation, multisensory integration, and certain aspects of perception (8-14). With regard to sensory reactivity, these features are frequently parsed into three specific behavioral “response patterns”: *hyperreactivity* (HYPER; i.e., excessive and/or defensive reactions to stimuli that most individuals find innocuous), *hyporeactivity* (HYPO; i.e., diminished or absent responses to sensory stimuli that most individuals would respond to), and *sensory seeking* (SEEK] i.e., unusually strong fascination with or craving of sensory stimulation, often accompanied by repeatedly seeking out specific sensory inputs (15-17)). Notably, these response patterns are not mutually exclusive, and many individuals express behaviors characteristic of multiple sensory response patterns, even within the same modality. Sensory reactivity differences are extremely common in autistic individuals: the point prevalence of a child displaying differences in any of the three response patterns (i.e., HYPER, HYPO, or SEEK in any modality) was recently estimated to be 74% using large-scale population-based data from the Autism and Developmental Disabilities Monitoring Network (18), and 70.9–88.3% of autistic youth in two large samples (from the United States and Australia, respectively) were determined to have sensory reactivity differences of at least “mild” severity (19, 20).

Although sensory reactivity differences are prevalent in many childhood-onset neurodevelopmental and neuropsychiatric conditions (e.g., (attention deficit hyperactivity disorder [ADHD], anxiety, obsessive-compulsive disorder, Tourette syndrome, Williams syndrome (21-26)), and all of these clinical groups can be differentiated from neurotypical controls in terms of sensory reactivity differences (see also (27)), a recent meta-analysis suggests that autistic individuals demonstrate higher average levels of HYPER (with findings mixed and inconclusive for HYPO and SEEK) when compared to individuals with other clinical conditions (28). Moreover, many qualitative and quantitative studies have linked specific sensory features of autism to functional impairment, reduced activity participation, and lower quality of life (e.g., (29-39), further emphasizing the importance of research into the sensory aspects of the autism phenotype. However, it is worth noting that not all sensory features of autism are inherently impairing or pathological, and some (particularly within the SEEK domain) are viewed positively by autistic people themselves (40-48).

Although recognition of the sensory features of autism has grown noticeably in recent years (28), relatively little published research in this area has evaluated structural relationships between different domains of sensory reactivity or tested the validity of existing theoretical subdimensions to describe this aspect of the autism phenotype (e.g., (49-54). The majority of studies examining sensory features in autism have utilized caregiver-report questionnaires such as the Sensory Profile (SP (55, 56)), the Sensory Experiences Questionnaire (SEQ (57-59)), and the Sensory Processing–3 Dimensions: Inventory (SP-3D:I (60, 61)), which are most often scored by generating supra-modal (i.e., combining multiple sensory modalities) HYPER, HYPO, and SEEK “response pattern scores” that aggregate items within a single response pattern across all assessed sensory modalities. Although these supra-modal constructs are consistent with the major conceptual models of sensory features (62-64), empirical support for the practice of combining responses to stimuli across multiple sensory modalities into a single “overall response pattern [HYPER, HYPO, or SEEK]” construct (as would be operationalized by a total score on all HYPER items, for instance; see (65)) is relatively limited. When examining the factor structures of existing sensory questionnaires, models that consider supra-modal response pattern factors in isolation tend to be inadequate, demonstrating very poor overall fit to empirical data (e.g., (66). Thus, in order to successfully explain the factor structure of the HYPER, HYPO, and SEEK constructs, previous studies have needed to utilize more complex models that include not only *supra-modal* response pattern factors but also *modality-specific* response pattern factors that account for the additional shared (co)variance between items within a given sensory modality (e.g., (49,52,67). As these models represent bifactor structures with variance attributable to both supra-modal and modality-specific constructs (68,69), summed supra-modal response pattern scores may only be clearly interpretable as measures of HYPER, HYPO, or SEEK if the strength of the modality-general factor is much stronger than the modality-specific factors (69-72).

In contrast to studying HYPER, HYPO, and SEEK at the supra-modal level, a minority of studies (e.g., (25,73-80) have investigated these sensory constructs in a *modality-specific* manner by calculating response pattern scores that are limited to a single sensory modality (e.g., (“Auditory HYPER,” which reflects the sum score of only the HYPER items within the Auditory modality). As psychophysical and neural measures of sensory function (e.g., (detection thresholds, psychometric function parameters, evoked potential amplitudes) are frequently limited to a single sensory modality, some researchers theorize that the modality-specific subconstructs represented by these measures will correlate more strongly with sensory reactivity measures that are limited to that same sensory modality rather than collapsed across modalities (e.g., (visual evoked potential amplitudes may be expected to correlate moreso with a measure of Visual HYPER than with general HYPER). To our knowledge, studies to date have not formally tested these hypotheses to determine whether or not modality-specific response pattern scores demonstrate any empirical advantages over conceptually broader supra-modal response pattern scores when correlated with psychophysical or neurophysiological measures of sensory function.

Determining the most appropriate “level of analysis” (supra-modal versus modality-specific versus some combination of the two) for these sensory constructs has major implications for other areas of sensory autism research, as this decision will impact whether modality-specific or supra-modal sensory constructs are assessed by diagnostic/phenotyping instruments, targeted by clinical interventions, correlated with other individual differences, explained with neuroscientific or psychological models (e.g., (multiple forms of sensory reactivity having a shared underlying mechanism or cause versus separate mechanisms [or even multiple mechanisms] being proposed for each form of sensory reactivity), and even incorporated into the diagnostic criteria for autism. Thus, additional research is needed to more conclusively determine whether sensory reactivity differences in autism are most appropriately studied at the level of a response pattern score (HYPER, HYPO, or SEEK) combined across modalities (e.g., (28,65,81-84), at the level of modality-specific response pattern scores (e.g., (74,76-79,85), or some combination of the two (e.g., (interpreting both types of scores; favoring one level of analysis at different points in a study based on the research question or the specific construct(s) being studied).

## Purpose

To address this critical gap in research on sensory features in autism, the present study sought to quantitatively investigate the latent structure of caregiver-reported sensory features across a large and heterogeneous group of autistic children. By pooling data from multiple independent research groups and the National Database for Autism Research (NDAR (86)), we compiled a cohort of several thousand autistic children to be analyzed within the methodological framework of integrative data analysis (IDA (87, 88)). The IDA approach has recently gained popularity within autism research, going beyond small sample studies to yield insights about the latent structure of core and associated autism features (54, 89-94), the psychometric properties of widely-used measures (54, 91, 95-100), and the associations between autism features and other related clinical and demographic variables (92, 98, 101-103). Utilizing modern psychometric techniques such as item response theory (IRT (104, 105)) and bifactor modeling (68, 69, 106), the current IDA sought to rigorously evaluate the latent structure of sensory features in autism across multiple measures. Our specific aims were to:

- derive psychometrically sound metrics of HYPER, HYPO, and SEEK within modalities;
- derive psychometrically sound supra-modal metrics of HYPER, HYPO, and SEEK;
- evaluate whether modality-specific HYPER, HYPO, and SEEK metrics (e.g., (Auditory HYPER, Tactile HYPO, Visual SEEK) provide added-value to the supra-modal metrics; and
- estimate meta-analytic associations between psychometrically derived sensory constructs and other clinical and demographic variables in the autistic population.

## Methods

### Participants

Data used in the current investigation were obtained from nine separate research groups within the Autism Sensory Research Consortium (https://tinyurl.com/ASRCoverview): University of North Carolina (*n* = 104), Vanderbilt University Medical Center 1 [PI: CJC] (*n* = 181), University of California San Francisco (*n* = 35), Syracuse University (*n* = 55), University of California Los Angeles (*n* = 67), Thomas Jefferson University (*n* = 93), University of Reading (n = 37), Kennedy Krieger Institute (*n* = 47), and Vanderbilt University Medical Center 2 [PI: TGW/MTW] (*n* = 114). Although no systematic review of the broader literature was undertaken, the included cases represented the full population of individual-participant data (including unpublished data) gathered by researchers within the Autism Sensory Research Consortium that could legally be shared with the first author’s institution. These data were further pooled with (a) all eligible non-overlapping data available in NDAR (*n* = 741 [15 collections; NDAR study 1160]), (b) data from a large online cohort including participants drawn from the Kennedy Krieger Institute-based Interactive Autism Network (IAN (107)) and various statewide and local autism advocacy groups (referred to as the Sensory Experiences Project [SEP] sample; *n* = 1285 (19)), and (c) data from individuals recruited from Simons Powering Autism Research for Knowledge (SPARK (108)) research match (project number RM0035_Woynaroski; *n* = 1107), resulting in a total of 3866 unique participants across all data sources (see Supplemental Table S1 for individual sample demographics). In accordance with IRB approved protocols for each primary study, informed consent was obtained from parents or legal guardians of each participant, and when relevant, assent was obtained from participants as well at the time of data collection. The institutional review board at Vanderbilt University Medical Center approved the secondary analysis of pooled data from these studies.

All participants included in the current study were between the ages of 3 years 0 months and 18 years 0 months and had clinical diagnoses of autism spectrum disorder according to DSM-5 criteria (or equivalent DSM-IV-TR diagnoses). Notably, we chose to restrict our analyses to autistic children as a way of protecting against both non-normal latent trait distributions and differential item functioning according to autism diagnostic status (109, 110). An additional criterion for inclusion in the current study was the accessibility of cross-sectional item-level data from one of the following sensory questionnaires: the Sensory Profile 1 (SP1 (55)), the Short Sensory Profile 1 (SSP1 (111)), the Sensory Profile 2 (SP2 (56)), the Short Sensory Profile 2 (SSP2 (56, 112)), the Sensory Experiences Questionnaire, version 2.1 (SEQ-2.1 (57, 113)), or the Sensory Experiences Questionnaire, version 3.0 (SEQ-3.0 (49,58)). Although other caregiver questionnaires such as the SP-3D:I were originally considered for inclusion in analyses as well, they were ultimately not included due to a very small number of individuals in our dataset (< 7% of the sample) with usable data on these measures. Broader inclusion/exclusion criteria for participation in contributing studies varied across samples; no participants represented in extant datasets were excluded from the current study due to any additional clinical characteristics (e.g., (language level, cognitive skills/IQ), demographic factors (with the exception of chronological age < 3 or > 18), co-occurring medical/psychiatric conditions, or receipt of specific interventions/services.

### Constructs And Measures

All participants in the current study had usable data on one or more of the primary study questionnaires, including the SP1/SSP1, SP2/SSP2, SEQ-2.1, or SEQ-3.0, and these measures were used to operationalize the sensory (sub)constructs of interest (see Supplemental Methods for additional details regarding the chosen sensory questionnaires and their use in the autistic population). In the current study, analogous items on the SP1 and SP2 (and their short forms) as well as analogous items on the SEQ-2.1 and SEQ-3.0 were combined into single items for the purpose of cross-dataset analysis. Notably, as the SP1/SSP1 are scored in the opposite direction of the remaining questionnaires, these measures were reverse-scored (i.e., such that scores of “5” represent *more frequent* behaviors) in order to keep item-level scoring consistent between all items in the study.

In addition to measures of sensory features, we also examined a number of putative demographic and clinical correlates, including age, sex at birth, cognitive ability, adaptive behavior, core autism features, and co-occurring psychiatric symptoms. Cognitive ability was assessed using verbal, nonverbal, and full-scale intelligence quotients (VIQ, NVIQ, and FSIQ, respectively [derived from many instruments]), their developmental quotient (DQ) analogs, and a binary indicator of intellectual disability status (FSIQ < 70 or NVIQ < 70 if no FSIQ available). Adaptive behavior was measured via summary scores (Communication [COM] domain, Daily Living Skills [DLS] domain, Socialization [SOC] domain, and Adaptive Behavior Composite [ABC]) from the Vineland Adaptive Behavior Scales (VABS (114)), including the first, second, and third editions of the measure. Core autism features were assessed using the Social Responsiveness Scale –School Age (SRS (115, 116)) total raw score, as well as the Repetitive Behavior Scale–Revised (RBS-R (117)) repetitive sensory motor (RSM), self-injurious behavior (SIB), and “ritualistic/sameness/compulsive” behavior (RSC) subscales (the latter being the sum of the RBS-R ritualistic/sameness and compulsive subscales due to a high intercorrelation between the two in the current sample; see Supplemental Methods). Co-occurring psychiatric symptoms (based on multiple measures) were summarized using the trait domains of “internalizing symptoms” (INT), “externalizing symptoms” (EXT), and “total psychiatric symptoms” (TOT), as well as features of ADHD (ADHD). See Supplemental Methods for additional information on the measures and scores used in the current study.

### Sensory Item Selection

**Figure 1:**
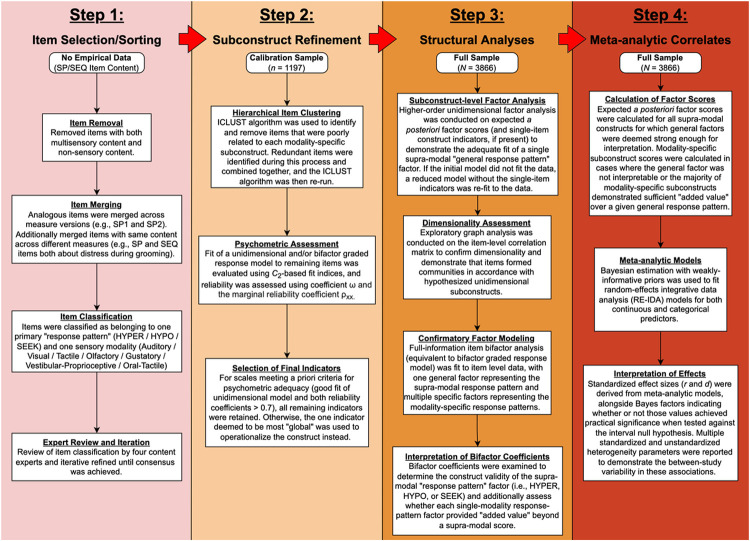
Schematic Diagram with Overview of Study Methodology

Before statistical analyses were undertaken, items from the four primary sensory questionnaires (SP1/SSP1, SP2/SSP2, SEQ-2.1, SEQ-3.0) were first subjected to a qualitative review by the first author to remove items that were multisensory in nature (e.g., (SEQ-3.0 item 95: *“How often does your child avoid playing with toys or novel objects that make a lot of noise and light up at the same time?”*) or appeared to assess non-sensory behaviors (e.g., (SSP1 item 27: *“Has difficulty paying attention.”*). The remaining items were then sorted into modality × response pattern “sensory subconstructs” (e.g., (Auditory HYPO) by the first author, and this sorting process was reviewed iteratively by four additional experts (authors RS, GTB, CJC, and TGW) until a consensus classification was reached. See Fig. 1 and Supplemental Methods for additional information on this process. A full list of items and their theoretical classifications can be found in Supplemental Table S2.

## Data Analysis

### Sensory Subconstruct Refinement and Empirical Item Removal

The first aim of this study was to develop psychometrically sound indicators of each sensory subconstruct (i.e., the combination of modality and response pattern, e.g., Visual HYPO, Auditory HYPER, Tactile SEEK). In order to develop these single-subconstruct scales, we started with all items theoretically classified as belonging to that subconstruct and empirically removed items until the resulting set of items conformed to a unidimensional structure, as defined below. In doing this, we aimed to generate a set of well-differentiated sensory constructs that could then be fit to a bifactor model (with a general factor representing the supra-modal response pattern and specific factors for each modality-specific subconstruct), allowing us to test the hierarchical structure of each sensory response pattern without poorly-related items inflating estimates of general factor saturation.

For the subconstruct refinement portion of the study, we opted to use data only from the subset of autistic children who provided data on both (a) one version of the SP and (b) one version of the SEQ (*n* = 930). However, as relatively few individuals in this pre-defined group of 930 had completed the SP2 or SSP2 (*n* = 26), we expanded this exploratory sample to encompass all other children in the full sample who had completed any version of the SP2 (*n* = 267). This resulted in a final sample of 1197 individuals, which we refer to as the “calibration sample”.

All data analyses were conducted in the R statistical computing environment, version 4.1.0 (118). Subscale item refinement was conducted in the calibration sample using an iterative process based on hierarchical item clustering with the ICLUST algorithm (119, 120), as implemented in the *psych R* package (121) (see Supplemental Methods for additional details). Once no more items met the criteria for removal, we evaluated the resulting scale for unidimensionality and reliability by fitting it to a graded response model (GRM (122); a type of IRT model) and assessing that model for global fit and composite score reliability (see Supplemental Methods for model specifications and specific psychometric criteria used to judge fit). Poor model fit or low reliability was followed up with examination of local misfit and iterative item removal with the goal of further improving model fit. In cases where a single-subconstruct scale demonstrated inadequate unidimensionality and/or insufficient reliability that could not be corrected by further item removal, that construct was deemed not sufficiently measurable with a subscale, and the subconstruct of interest was then operationalized using the single item from the available pool deemed most global or nonspecific by the first author. This process was repeated for each subconstruct until there was at least one unidimensional scale or single item available to assess all subconstructs of interest for the current investigation.

### Structural Evaluation of Supra-modal Sensory Response Pattern Constructs

The second aim of the present study was to better understand the structure of supra-modal sensory “response pattern” constructs (i.e., HYPER, HYPO, SEEK). After we had generated viable indicators (either a unidimensional scale or a single item) for each modality-specific subconstruct within a given response pattern (e.g., (Visual, Auditory, Tactile modalities within HYPER), we then sought to examine the higher-order factor structure of these indicators by conducting a scale-level factor analysis of these scores (or in some cases, items) in our full sample of 3866 autistic children (see Supplemental Methods for full model specifications and fit criteria). If these models did not demonstrate adequate fit, we subsequently re-fit the models without the single-item sensory constructs (i.e., only conducting a CFA of the continuous EAP scores). If either unidimensional factor model (i.e., the original model or the re-fit model) fit the covariance structure of the subscales adequately, the response pattern of interest was deemed to have some degree of evidence for a supra-modal factor.

### Bifactor Modeling of Sensory Constructs

To further assess the validity of the supra-modal HYPER, HYPO, and SEEK constructs, we additionally examined the *item-level* latent structure for each response pattern using confirmatory bifactor IRT models. This was done using the same pool of items found to produce the best-fitting subscale-level model (i.e., if single-item constructs were removed from the subscale-level CFA, they were not included in this stage of the structural evaluation). Approximate simple structure (i.e., items loading on expected modality factors) was first confirmed using exploratory graph analysis (EGA (123, 124); see Supplemental Methods for technical details), and once this structure was confirmed, item-level data were fit to a bifactor GRM (125, 126). Model fit was assessed with the same criteria for adequate fit as used for the unidimensional GRMs (see Supplemental Methods).

After confirming the adequate fit of a bifactor GRM, bifactor model-based indices (70, 71, 127) were calculated to determine the appropriateness of interpreting supra-modal response pattern scores. Bifactor indices that were examined included coefficient omega total (ω_⊤_; model-based total score reliability), coefficient omega subscale (ω_S_; model-based subscale score reliability), coefficient omega hierarchical (ω_H_; proportion of variance in total score accounted for by general factor), coefficient omega hierarchical subscale (ω_HS_; proportion of variance in subscale score accounted for by general factor), explained common variance of the general factor (*ECV*_G_), and explained common variance of the specific factor for each subscale (*ECV*_SS_). We defined bifactor structures with ω_H_ ≥ .80 (70) or the combination of ω_H_ ≥ .70 and *ECV*_G_ ≥ .60 (128) as demonstrating evidence of a strong general factor, and thus, a valid and interpretable higher-order construct (129).

A third aim of the study was to determine whether modality-specific subconstruct scores provide substantial “added value” over the supra-modal response pattern scores in characterizing the sensory features of autism. In the current study, we specifically addressed this question using recently-proposed psychometric decision rules based on combinations of bifactor model-based statistics (127). Modality-specific subconstruct scores (i.e., the set of items representing one subconstruct such as Tactile SEEK) with low reliability (ω_S_ ≈ .60) were considered to have sufficient added value with ω_HS_ ≥ .25 or *ECV*_SS_ ≥ .45; sensory subconstructs with high reliability (ω_S_ ≈ .80), were considered to have sufficient added value with ω_HS_ ≥ .20 or *ECV*_SS_ ≥ .30 (127). With these analyses, we could determine whether the HYPER, HYPO, and SEEK constructs should be interpreted (a) only at the supra-modal level (i.e., consideration of HYPER as a unitary construct), (b) only for individual modalities (i.e., consideration of Auditory HYPER and Tactile HYPER as distinct modality-specific subconstructs), or (c) as a combination of the two (analogous to interpretation of FSIQ as well as VIQ/NVIQ on an intelligence test).

### Demographic and Clinical Correlates of Sensory Reactivity

#### Modeling Procedures.

The fourth aim of this study was to evaluate correlations between sensory reactivity and demographic and clinical characteristics. Based on the previous bifactor analyses, EAP factor scores were calculated for all interpretable modality-specific subconstructs and supra-modal constructs (130) and then examined using random-effects IDA models (87), which use random effects to account for the heterogeneity of effect sizes among the different study samples. Correlates examined in these models included chronological age, sex (female versus male), cognitive scores (VIQ/VDQ, NVIQ/NVDQ, and FSIQ/FSDQ), intellectual disability status (which was defined as FSIQ < 70 [or NVIQ < 70 in cases where FSIQ was missing], excluding DQ scores), psychiatric symptom scores (internalizing symptoms, externalizing symptoms, total psychiatric symptoms, ADHD features), VABS scores (COM, DLS, SOC, ABC), and core autism features (SRS total score, RBS-R scores [RSM, SIB, and RSC]). Models were not fit for combinations of sensory outcomes and putative correlates with fewer than 100 observed cases. The random-effects IDA models were specified as Bayesian hierarchical linear models with a standardized subconstruct score regressed on the correlate of interest; additional random intercept and slope terms were also added to account for between-study mean differences in the outcome and effect sizes. Weakly-informative priors were placed on all model parameters. These models were fit using the *brms* R package (131, 132). Supplemental Table S3 contains additional details regarding IDA model and prior specification, including computational specifications.

To summarize the strength of each IDA-based association, standardized effect sizes (Cohen’s *d* for categorical predictors and the linear correlation *r* for continuous predictors) were calculated based on the standardized regression slopes, and posterior distributions were summarized using the median and 95% highest-density credible interval (CrI (133)). Effect size posterior distributions were tested against interval null hypotheses that the effects were too small to be practically significant (*d* = [−0.2, 0.2] and *r* = [−1, .1], respectively (134)). It is important to note that such interval null-hypothesis tests have been found to demonstrate substantially lower false-positive rates that traditional frequentist tests of a point-null hypothesis (135), thereby playing the role of a multiple testing correction in the current study (though see also (136, 137) for Bayesian perspectives on multiple testing). These interval null hypotheses were assessed using a Bayesian hypothesis testing procedure based on the region of practical equivalence (ROPE (133)) and the ROPE Bayes factor (*BF*_ROPE_ (138, 139)). From the posterior distribution of effect sizes, we calculated the following indices: (a) *P*_ROPE_, the posterior probability that the null hypothesis is true (i.e., the summary effect size is too small to be practically meaningful), (b) log(*BF*_ROPE_), a measure of evidence that the summary effect size falls within versus outside the ROPE. Values of log(*BF*_ROPE_) greater than 1.1 and 2.3 (i.e., log(3) and log(10)), respectively, provide moderate and strong evidence that the true effect size lies outside the ROPE (i.e., evidence that the effect is large enough to be practically meaningful), and log(*BF*_ROPE_) values less than −1.1 and −2.3, respectively, provide moderate and strong evidence that the effect size lies within the ROPE (i.e., the effect is practically equivalent to zero). If log(*BF*_ROPE_) value lies between −1.1 and 1.1, the evidence for or against the interval null hypothesis is deemed inconclusive (140). These Bayesian indices were calculated using the *bayestestR* R package (141).

From each IDA model, we also calculated several heterogeneity indices, including τ^2^ (the variance of the random slope parameter in standard deviation units), *I*^2^ (the percentage of variance in the slope parameter due to between-study heterogeneity), and the intraclass correlation coefficient (ICC; the proportion of total variance accounted for by both the random slope and intercept terms). Lastly, we calculated the 95% prediction interval of *r* or *d* (142, 143), which includes the range of values likely to be sampled from a new study of similar size to the ones included in the current analyses.

## Results

Demographic and clinical characteristics of the sample are displayed in Table 1, and characteristics of each contributing sample can be found in Supplemental Table S1. Children in the combined sample had a mean age of 8.41 (*SD* = 3.36) years and were predominantly male (79.5%) and White/Caucasian (75.5%; 84.4% of those with non-missing data). The mean full-scale IQ/DQ of children with available data (*n* = 1028) was 92.1 (*SD* = 24.5), with FSIQ/DQ scores ranging from 12.0–153.0 (FSIQ: *M ± SD* [min−max] = 98.98 ± 19.59 [32.0–153.0]; FSDQ: *M ± SD* [min−max] = 59.08 ± 18.45 [12.0–124.0]).

## Sensory Subconstruct Refinement

Results of the scale refinement process for each subconstruct are presented in Table 2. Within the HYPER domain, items were originally sorted into six modalities (Auditory, Visual, Tactile/Somatosensory, Olfactory, Gustatory, and Vestibular/Proprioceptive [Movement]), each of which produced a unidimensional subconstruct scale that met our a priori requirements for reliability and model fit. Notably, during the scale refinement process, adequately-fitting bifactor structures were also found for both Auditory HYPER (7 items, 1 specific factor; Supplemental Table S4) and Tactile HYPER (13 items, 4 specific factors; Supplemental Table S5), meeting all of our a priori psychometric requirements aside from unidimensionality. For these two sensory subconstructs, unidimensional scales (4 items each) were utilized in the cross-modality structural analysis of HYPER, although subsequent single-modality models were built using the more reliable general factor scores from these bifactor models, as the latter included a broader item pool that was inclusive of more information about the sensory subconstruct of interest.

Within the HYPO domain, items were originally sorted into five modalities (Auditory, Visual, Tactile/Somatosensory, Olfactory, and Gustatory), although two of these modalities (Olfactory and Gustatory) ultimately contained fewer than the minimum of three items needed for a subconstruct scale. Thus, these two subconstructs were operationalized with single items rather than multi-item composites (Olfactory: SEQ-3.0 item 69: *“How often does your child seem to be unaware of strong or unpleasant smells that most other people notice?”*; Gustatory: SEQ-3.0 item 74: *“How often does your child have trouble distinguishing between different types of tastes or flavors?”*). From the Auditory HYPO items, a three-item composite met our criteria for an adequate subconstruct scale; however, after examining the content of the items (Supplemental Table S2), it was noted that these three items measured hyporeactivity to speech in particular, rather than auditory stimuli more generally. Thus, to ensure that reactivity to both speech and nonspeech auditory stimuli were both captured in the analysis of HYPO constructs, an additional single item (SEQ-2.1 item 4/SEQ-3.0 item 4: *“How often does your child ignore or tune-out loud noises?”*) was included to capture non-speech Auditory HYPO. Similarly, although a reliable three-item scale could be derived from the Tactile HYPO items, this scale only contained items assessing hyporeactivity to pain and temperature. Thus, an additional single item (SP1 item 46/SP2 item 26: *“Doesn't seem to notice when face or hands are messy”*) was chosen as the single indicator for Tactile HYPO unrelated to pain or temperature. Lastly, although the Visual HYPO scale contained three items and fit a unidimensional model adequately (SRMR = .024), the scale demonstrated subpar reliability (ω = .656, ρ_xx_ = .631) and, thus, was not retained. We therefore used a single item (SEQ-2.1 item 10/SEQ-3.0 items 22/23: *“Is your child slow to notice new objects or toys in the room, or slow to look at objects that are placed or held near him/her?”*) to capture Visual HYPO in our structural analyses.

Within the SEEK domain, items were originally sorted into seven modalities (Auditory, Visual, Tactile/Somatosensory, Olfactory, Gustatory, Oral Tactile, and Vestibular/Proprioceptive [Movement]). Notably, based on discussions during the item sorting, the SEEK response pattern encompassed the additional modality of Oral Tactile, which contained items from the SP/SEQ “Taste/Smell” sections that specifically describe a child mouthing or licking nonfood objects without necessarily seeking out the taste of those objects. Of the SEEK subconstructs, unidimensional scales were derived from four (Visual, Tactile, Oral Tactile, and Vestibular/Proprioceptive [Movement]). A three-item Auditory SEEK scale was found to demonstrate adequate fit (SRMR = .010) but subpar reliability (ω = .623, ρ_xx_ = .729); thus, it was replaced with a single-item indicator of Auditory SEEK (SEQ-2.1 item 36a/SEQ-3.0 item 7: *“How often does your child seem fascinated with sounds?”*). Additionally, three-item Olfactory and Gustatory SEEK scales demonstrated inadequate model fit (Olfactory: SRMR = .045; Gustatory: SRMR = .044); therefore, these constructs were replaced with single items as well (Olfactory: SEQ-2.1 item 36c/SEQ-3.0 item 67: *“How often does your child seem fascinated with particular smells?”*; Gustatory: SEQ-3.0 item 64: *“How often does your child crave foods with a strong taste or flavor (such as spicy sour, or bitter foods)?”*).

## Structural Evaluation Of Supra-modal Sensory Constructs

### HYPER

To examine the higher-order structure of HYPER constructs, expected *a posteriori* (EAP) factor scores were derived from unidimensional models for each modality (i.e., Auditory, Visual, Tactile/Somatosensory, Olfactory, Gustatory, and Vestibular/Proprioceptive) and fit to a unidimensional CFA model using MLR estimation. This CFA model fit the data adequately (χ^2^(9) = 75.3, CFI = .974, RMSEA = .044, SRMR = .024), and all single-modality factor scores loaded with moderate to moderately large magnitudes onto the higher-order HYPER factor (λ = 0.445–0.638). The item-level latent structure of the 23 HYPER items was then examined using EGA, with the community structure recreating the six hypothesized modality-specific factors. A bifactor GRM with specific factors for each modality demonstrated largely adequate global fit (*C*_2_(228) = 380.9, TLI_C2_ = 0.985, RMSEA_C2_ = .036, SRMR = .053); thus, the bifactor coefficients from this model were interpreted to determine the strength of the general HYPER factor.

**Figure 2:**
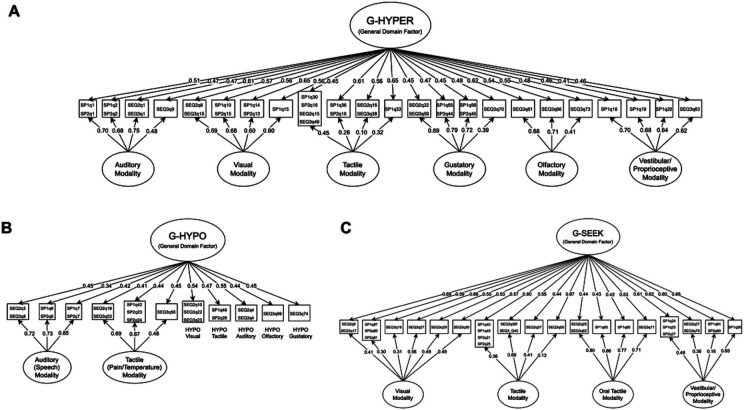
Path Diagrams of Final Bifactor Models for (A) Hyperreactivity (HYPER), (B) Hyporeactivity (HYPO), and (C) Sensory Seeking (SEEK) Sensory Response Patterns

Factor loadings and bifactor coefficients from the HYPER model are presented in Table 3 and Fig. 2A. Coefficient omega total indicated that the overall HYPER sum score was highly reliable (ω_⊤_ = .988), and coefficient omega hierarchical indicated that the general factor saturation was sufficient to justify interpretation of the total score (ω_H_ = .800). However, despite the adequate ω_H_ value, the general factor explained a relatively low proportion of common variance (*ECV*_G_ = .436, i.e., 43.6%), suggesting that modality-specific subconstruct factors were responsible for a slight majority (56.4%) of reliable common variance in HYPER items (see also *ECV*_G_ and *ECV*_SS_ values in Table 3 for the proportions of common variance in each subconstruct accounted for by general and specific factors, respectively). Moreover, based on the guidelines of Dueber & Toland (2021), five of six modality-specific HYPER subconstructs (all except Tactile/Somatosensory) demonstrated added value beyond that provided by the total HYPER score (i.e., ω_S_ > .80 and *ECV*_SS_ ≥ .30 Table 3). Thus, we concluded that both the HYPER total score and subconstruct scores were interpretable. EAP scores for both the HYPER general factor (calculated from the bifactor GRM) and modality-specific HYPER subconstruct factors (calculated from unidimensional GRMs or from bifactor GRMs in the case of Auditory and Tactile HYPER) were, therefore, examined in relation to demographic and clinical correlates.

### HYPO

To examine the higher-order structure of the HYPO constructs, we used WLSMV estimation to fit a CFA model to the EAP scores for the two multi-item HYPO constructs (Auditory-Speech and Tactile-Pain/Temperature), as well as five single-item HYPO indicators for each sensory modality (i.e., Auditory [non-speech], Visual, Tactile, Olfactory, and Gustatory). The CFA model fit the data adequately (χ^2^(14) = 87.7, CFI = .960, RMSEA = .037, SRMR = .038), and all standardized factor loadings were moderate in magnitude (λ = 0.416–0.516). The item-level latent structure of the 11 HYPO items was then examined using EGA, with the Walktrap algorithm indicating a two-factor solution with hyporeactivity to speech in one community and all other items in the second community. Although the hyporeactivity to pain/temperature items did not form their own community, this structure nevertheless provided support for the separation of HYPO pain/temperature and HYPO speech subconstructs. The 11 items were then fit to a bifactor GRM with one general factor and two specific factors (Speech HYPO and Pain/temperature HYPO); all subconstructs represented by single items were specified to load only onto the general factor.

Factor loadings and bifactor coefficients for the HYPO model are presented in Table 4 and Fig. 2B. This model demonstrated adequate fit on all indices other than SRMR (*C*_2_(38) = 58.6, TLI_C2_ = 0.987, RMSEA_C2_ = .033, SRMR = .065) and high total score reliability (ω_⊤_ = .905). However, additional bifactor coefficients did not support the interpretation of the general HYPO factor, which fell below both a priori guidelines for general factor saturation (ω_H_ = .611) and proportion of explained common variance (*ECV*_G_ = .413; see also *ECV*_G_ and *ECV*_SS_ values in Table 4 for the proportions of common variance in each subscale accounted for by general and specific factors, respectively). Moreover, both the Speech HYPO (ω_S_ = .929, ω_HS_ = 0.710, *ECV*_SS_ = .781) and Pain/temperature HYPO (ω_S_ = .845, ω_HS_ = .579, *ECV*_SS_ = .715) subconstructs demonstrated large proportions of specific-factor variance, indicating substantial added value over a general HYPO score. Thus, as HYPO subconstruct scores but not the supra-modal HYPO score met our guidelines for interpretability, only the Speech HYPO and Pain/temperature HYPO EAP scores (calculated from unidimensional GRMs) were examined in our analysis of clinical/demographic correlates.

### SEEK

To examine the higher-order structure of the SEEK constructs, we fit a CFA model to the Visual, Tactile, Oral Tactile, and Vestibular/Proprioceptive [Movement] EAP scores, as well as three single-item SEEK indicators for each additional sensory modality (i.e., Auditory, Olfactory, and Gustatory). The initial CFA model (WLSMV estimation) fit the data poorly (χ^2^(14) = 361.9, CFI = .896, RMSEA= .080, SRMR = .057); therefore, this model was not interpreted. We then fit a model that excluded the three single-item SEEK indicators, only examining the four EAP subconstruct factor scores. This CFA model (MLR estimation) demonstrated good fit to the data (χ^2^(2) = 13.7, CFI = .994, RMSEA = .039, SRMR = .013), and all factor loadings were moderate to moderately large in magnitude (λ = 0.439–0.685). The 18 SEEK items (excluding all single-item indicators) were then examined using EGA, with the resulting community structure recreating the hypothesized modality-specific subconstructs. These items were then fit to a bifactor GRM with one general factor and four modality-based specific factors.

Factor loadings and bifactor coefficients for the SEEK model are presented in Table 5 and Fig. 2C. The model demonstrated adequate fit for all indices other than SRMR (*C*_2_(117) = 232.9, TLI_C2_ = 0.982, RMSEA_C2_ = .044, SRMR = .058), and total score reliability was very high (ω_⊤_ = .969). Notably, the general factor saturation of this model was just below the a priori threshold for interpretability (ω_H_ = .799), and while the explained common variance was still relatively low (*ECV*_G_ = .531; see also *ECV*_G_ and *ECV*_SS_ values in Table 5 for the proportions of common variance in each subscale accounted for by general and specific factors, respectively), this suggested that a composite SEEK score could potentially be interpretable. However, as we excluded three modality-specific subconstructs that fit poorly in the higher-order SEEK CFA, the omega hierarchical estimate from the 18-item bifactor structure likely overestimated the true general factor saturation of a scale consisting of all seven modalities (i.e., the construct captured by the supra-modal score on the SP or SEQ SEEK composite). Thus, as this overestimate did not exceed the a priori-specified threshold for general factor interpretability, we chose to not interpret the SEEK general factor score. Supporting our decision to interpret SEEK at the single-modality level only, additional bifactor indices suggested that all four modality-specific SEEK subscale scores demonstrated added value over the total score (Visual: ω_S_ = .934, ω_HS_ = .686, *ECV*_SS_ = .747; Tactile: ω_S_ = .751, ω_HS_ = .255, *ECV*_SS_ = .380; Oral: ω_S_ = .840, ω_HS_ = .241, *ECV*_SS_ = .337; Movement: ω_S_ = .876, ω_HS_ = .287, *ECV*_SS_ = .344). Thus, only the EAP scores for the four SEEK subconstructs (calculated from unidimensional GRMs) were examined in our analysis of clinical/demographic correlates.

## Demographic And Clinical Correlates

### Modeling

**Figure 3:**
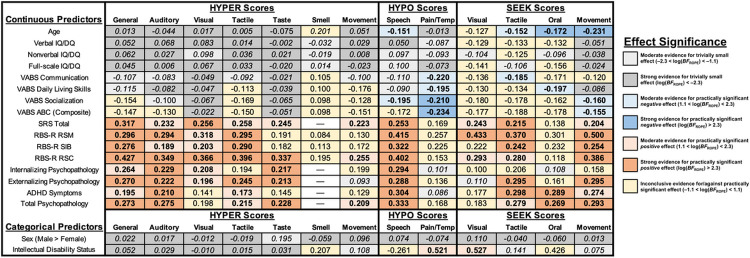
Meta-analytic Standardized Effect Sizes for Associations between Sensory Subconstructs and Putative Demographic and Clinical Correlates

Figure 3 displays a summary of all bivariate IDA model-based effect sizes estimating relations between EAP sensory scores and identified clinical and demographics correlates (i.e., *r* for continuous variables and *d* for binary variables). Additional model-based statistics, including effect size credible intervals, posterior probabilities of the interval null hypothesis (*P*_ROPE_), log(*BF*_ROPE_) values, predictive intervals, and heterogeneity estimates are presented in Supplemental Tables S6-S7. Of 240 correlations examined (with interval null hypothesis tests used to control the false-discovery rate; ,135), 47 (19.6%) demonstrated strong evidence for a practically significant effect (log(*BF*_ROPE_) > 2.3), 30 (12.5%) demonstrated moderate evidence for a practically significant effect (1.1 < log(*BF*_ROPE_) < 2.3), 70 (29.2%) demonstrated strong evidence for a trivially small effect (log(*BF*_ROPE_) < −2.3), and 27 (11.2%) demonstrated moderate evidence for a trivially small effect (−2.3 < log(*BF*_ROPE_) < −1.1). The remaining 66 correlations (27.5%) provided inconclusive evidence for or against the presence of a practically significant effect (−1.1 < log(*BF*_ROPE_) < 1.1). Additionally, of the 26 examined Cohen’s *d* values, two (7.7%) demonstrated moderate evidence for a practically significant effect (1.1 < log(*BF*_ROPE_ < 2.3), 17 (65.4%) demonstrated strong evidence for a trivially small effect (log(*BF*_ROPE_) < −2.3), four (15.4%) demonstrated moderate evidence for a trivially small effect (−2.3 < log(*BF*_ROPE_) < −1.1), and three (11.5%) were inconclusive (−1.1 < log(*BF*_ROPE_) < 1.1). Heterogeneity varied greatly between models (ICC range 0.012–0.823), with random effects typically accounting for approximately 10–20% of total score variance (Supplemental Tables S6-S7).

### Correlations with Demographic Variables

Caregiver-reported sensory reactivity demonstrated relatively few significant correlations with demographic variables (i.e., age, sex), and almost all log(*BF*_ROPE_) values for these models moderately or strongly favored the null hypothesis of a trivially small effect. However, younger age was significantly associated with higher Speech HYPO (*r*= − .151, CrI_95%_ [−.214, − .094]), as well as a higher degree of SEEK in the Tactile (*r*= − .152, CrI_95%_ [−.205, − .099]), Oral (*r*= − .172, CrI_95%_ [−.250, − .107]), and Vestibular/Proprioceptive [Movement] (*r*= − .231, CrI_95%_ [−.323, − .133]) modalities, though these effects were small in magnitude.

### Correlations with Cognition and Adaptive Functioning

No sensory variable demonstrated practically significant associations with VIQ, NVIQ, or FSIQ when measured continuously. However, moderate group differences were found between individuals with and without intellectual disability for both Pain/Temperature HYPO (*d* = 0.521, CrI_95%_ [0.083, 0.959]) and Visual SEEK (*d* = 0.527, CrI_95%_ [0.164, 0.875]) such that individuals with intellectual disability were reported to have higher scores on both constructs. Adaptive skills, as measured by multiple VABS subscales, demonstrated small yet practically significant negative associations with Pain/Temperature HYPO (VABS COM: *r*= − .220, CrI_95%_ [−.344, − .089]; VABS DLS: *r*= − .195, CrI_95%_ [−.323, − .076]; VABS SOC: *r*= − .210, CrI_95%_ [−.294, − .123]; VABS ABC: *r*= − .234, CrI_95%_ [−.342, − .115]), Speech HYPO (VABS SOC: *r*= − .195, CrI_95%_ [−.318, − .079]), Tactile SEEK (VABS COM: *r*= − .185, CrI_95%_ [−.293, − .078]), Oral SEEK (VABS DLS: *r*= − .197, CrI_95%_ [−.306, − .075]), and Movement SEEK (VABS SOC: *r*= − .160, CrI_95%_ [−.227, − .097]; VABS ABC: *r*= − .155, CrI_95%_ [−.220, − .088]).

### Correlations with Core Autism Features and Psychiatric Symptoms

Although not associated with cognitive or adaptive behavior scores to a practically meaningful degree, most subconstructs in the HYPER response pattern displayed small to moderate associations across domains of core autism features (SRS, RBS-R RSM/SIB/RSC), ADHD symptoms, and broad measures of caregiver-reported psychiatric symptoms; these relations ranged from small to large in magnitude (Fig. 3; Supplemental Table S6). Speech HYPO, Tactile SEEK, and Movement SEEK also demonstrated significant relations with the majority of caregiver-reported symptom domains. Other SEEK domains demonstrated somewhat more selective patterns of correlations, with Visual SEEK correlating significantly with core autism features (SRS, RBS-R RSM), and Oral Tactile SEEK correlating significantly with ADHD features and total psychiatric symptoms. Notably, the majority of non-significant correlations between sensory reactivity and core autism features or psychiatric symptoms were inconclusive rather than trivially small, and thus it is likely the case that many of the true population correlations do not fall within *r*= [−1, .1].

## Discussion

Despite the recent elevation of sensory reactivity differences to the status of a diagnostic criterion for autism (15, 16), there has been relatively little empirical work examining the underlying latent structure of these core sensory features within the autistic population (see also 49). By analyzing caregiver-reported sensory reactivity differences in a heterogeneous cross-sectional sample of nearly 4,000 autistic children, the current study sought to investigate the hierarchical structure of sensory hyperreactivity (HYPER), hyporeactivity (HYPO), and seeking (SEEK) across the full spectrum of children captured under the label of autism spectrum disorder. Utilizing modern psychometric techniques, we developed structural models of HYPER, HYPO, and SEEK in individual sensory modalities, subsequently testing whether each construct is most appropriately studied at the level of a single supra-modal sensory response pattern (e.g., (an overall SEEK score) or separately for each modality within the sensory response patterns (e.g., (separate scores for Visual SEEK, Auditory SEEK, and Tactile SEEK). Of the three sensory response patterns included within current autism diagnostic criteria (15, 16), only HYPER demonstrated unambiguous evidence of an interpretable supra-modal construct, whereas supra-modal HYPO scores as currently operationalized were found to have limited construct validity. The evidence for supra-modal SEEK scores was more ambiguous, as we were unable to generate an adequately-fitting higher-order model of SEEK that included all relevant sensory modalities, but once modalities measured with only single items (i.e., Auditory, Olfactory, and Gustatory) were removed, the model containing the four remaining modalities (Visual, Tactile, Oral Tactile, and Movement) demonstrated an adequately fitting higher-order structure and borderline acceptable general factor saturation. Although our findings did not conclusively support the construct validity of a SEEK composite that includes all seven standard modalities (i.e., those operationalized by the SP or SEQ SEEK response pattern scores), the more limited “General SEEK” construct described here (consisting of only Visual, Tactile, Oral Tactile, and Movement items) may be a useful supra-modal aspect of the sensory autism phenotype if replicated in future studies. Additionally, irrespective of the construct validity of supra-modal scores, nearly all modality-specific sensory subconstructs demonstrated added value over and above their respective response pattern scores, indicating that modality-specific HYPER, HYPO, and SEEK scores are able explain additional individual differences in sensory reactivity to a greater degree than a single supra-modal HYPER, HYPO or SEEK score. These findings have implications for researchers interested in characterizing, explaining, or intervening on sensory reactivity in autistic individuals, as they suggest that some of the supra-modal response pattern scores used in these areas may have previously unrealized psychometric limitations. HYPER, HYPO or SEEK scores that are limited to one modality (i.e., single-modality subconstruct scores) could potentially prove advantageous in some contexts, although additional research is necessary to determine the extent to which these measures demonstrate incremental clinical or practical utility over established supra-modal HYPER, HYPO, or SEEK scores.

The current study also provided a wealth of information about the measurement of sensory reactivity in autistic youth based on caregiver-report questionnaires. Using the most frequently employed caregiver-report sensory measures in the autism literature (the SP and SEQ), we attempted to generate unidimensional scales to operationalize each combination of modality × response pattern as its own unique sensory subconstruct. However, based on a priori psychometric criteria, we were unable to generate acceptable unidimensional scales for three of five HYPO modalities (Visual, Olfactory, Gustatory) and three of seven SEEK modalities (Auditory, Olfactory, Gustatory), necessitating the use of single-item indicators of these subconstructs in later structural analyses. Moreover, within the HYPO domain, the two subconstructs that did produce sufficiently reliable scales reflected fairly specific subsets of the total item pool (e.g., (hyporeactivity to pain and temperature rather than all somatosensory stimuli), suggesting that they did not fully operationalize the “Auditory HYPO” and “Tactile HYPO” constructs that we had originally intended to measure. Thus, despite broadband sensory reactivity measures such as the SP, SEQ, and SP-3D:I typically including HYPER, HYPO, and SEEK items for each sensory modality, a sizable minority of “modality × response pattern” subconstructs demonstrated inadequate construct validity within this large autistic sample. In the current study, is unclear whether this finding stems from instrument-specific measurement issues (i.e., inadequate construct coverage within the specific questionnaires from which items were drawn) as opposed to more general issues regarding the theoretical definition of the construct or its ability to be reliably operationalized as a set of observer-reported questionnaire items. As both sets of issues are likely to contribute in different cases, we suggest some sensory subconstructs (particularly within the HYPO and SEEK domains) are (a) underrepresented in existing questionnaires (i.e., more questions are needed), (b) poorly defined (e.g., (Visual HYPO items have unclear relations with specific aspects of visual perception), (c) difficult for caregivers to report on reliably (e.g., (few indicators of Gustatory SEEK are present in most children, limiting the pool of potential items available to capture this construct), and/or (d) of potentially limited theoretical relevance when predicting clinical outcomes (e.g., (Olfactory HYPO may be unlikely to substantially influence the expression of other core features of autism). Future work should attempt to evaluate which, if any, of these poorly operationalized sensory subconstructs are relevant to autism research and clinical practice and if so, how they can be reliably measured.

As the majority of work examining sensory constructs in both autism and other clinical populations has utilized supra-modal scores from the SP, SEQ, SP-3D:I, or similar measures (e.g., (27,28,81,144,145), our findings signal the need for sensory autism research to broaden the ways in which sensory reactivity differences are characterized, potentially shifting away from the field’s nearly exclusive reliance upon supra-modal HYPER, HYPO, and SEEK scores for this purpose. Single-modality measures of HYPER, HYPO, and/or SEEK represent a viable alternative method of assessing these constructs and may be particularly useful when substantive research hypotheses include associations with other sensory constructs in a single modality (e.g., (tactile detection thresholds, visual evoked potentials). Moreover, there is a great need to develop more comprehensive measures of modality-specific HYPER, HYPO, and SEEK subconstructs, either by expanding upon the item banks employed in the current study, adapting questionnaires used in other fields (e.g., (146), or developing and validating entirely novel measures (e.g., (85). By more densely sampling each subconstruct of interest, these measures would ostensibly increase the reliability, validity, conceptual breadth, and clinical utility of modality-specific response pattern scales compared to the short and relatively general item pools currently included in longer broadband sensory measures. Importantly, we are not suggesting that researchers entirely abandon the study of supra-modal sensory constructs—particularly HYPER, for which we have found some empirical support for the supra-modal response pattern—as there is certainly value in the investigation of these higher-order constructs as well. Rather, in future studies where both supra-modal and modality-specific subconstruct scores could feasibly be interpreted, we strongly recommend that researchers use contextual factors to determine which “level of analysis” is most appropriate or informative to answer the substantive research question(s) at hand.

For researchers who do choose to characterize sensory reactivity at the single-modality level going forward, it is notable that the “level of analysis” chosen to study a problem will likely frame the ways in which sensory features of autism are conceptualized and studied more broadly as an aspect of autism’s heterogeneity (e.g., (147). In particular, when developing clinical interventions for sensory reactivity in autism, a focus on modality-specific sensory subconstruct outcomes may motivate clinicians and researchers to investigate the efficacy of intervention modalities that are more focused on specific subconstructs rather than sensory reactivity in general (e.g., (the use of sound generators to treat hyperacusis, a specific type of Auditory HYPER (148)). Personalized interventions that seek to assess an autistic child’s specific pattern of sensory reactivity differences and ameliorate challenges associated with each domain could also be assessed within this framework, using modality-specific assessments of each sensory response pattern to monitor the effectiveness of each putative “active ingredient” of the intervention. A shift in measurement practices will also allow researchers to associate these single-modality behavioral subconstructs with psychophysical and/or neurophysiological measures within a given modality (e.g., (74,75), informing theories of the neurocognitive underpinnings of certain types of sensory reactivity in autism (e.g., (39,149,150). Though we do not claim a single-modality perspective to be advantageous in all cases or for all research questions (particularly for those focused on “real-world” multisensory contexts), we believe that a greater diversity of theoretical approaches and frameworks within sensory autism research is needed to make optimal progress towards improving the lives of autistic people within this line of work.

In addition to our examination of the latent structure of sensory reactivity and assessment of evidence to support each “level of analysis,” we also employed random-effects integrative data analysis to estimate the meta-analytic associations between all sufficiently interpretable sensory subconstructs (i.e., General HYPER, six HYPER subconstructs, two HYPO subconstructs, and four SEEK subconstructs) and relevant demographic and clinical correlates (i.e., age, sex, cognitive abilities, adaptive functioning, core autism features, and co-occurring psychiatric symptoms). In general, the majority of associations were modest in size (with a few exceptions, e.g., large correlations between RBS-R RSM/Movement SEEK and RBS-R RSC/General HYPER), and Bayes factor tests indicated that many of the observed effects (particularly associations with age, sex, adaptive functioning, and cognitive abilities), were small enough to be practically equivalent to zero. Notably, none of the assessed sensory variables were significantly associated with sex or cognitive abilities as continuously quantified, although intellectual disability status was associated with moderately higher levels of Pain/temperature HYPO and Visual SEEK. Significant negative associations were also observed between certain HYPO/SEEK scores and adaptive behavior scores (with Pain/temperature HYPO demonstrating the most consistent associations); however, these effects were relatively small in magnitude (∣*r*∣s < .234).

In line with the classification of sensory reactivity differences as a core diagnostic criterion for autism classified under restricted/repetitive behaviors and interests, most sensory subconstructs correlated moderately with one or more of the RBS-R subscales (i.e., RSM [lower-order repetitive behaviors] and/or RSC [higher-order repetitive behaviors]). Notably, the largest summary effect observed in the current study was the correlation between Movement SEEK and the RBS-R RSM subscale, although this was likely driven to some extent by overlapping item content (e.g., (both SEQ 2.1 Item 27/SEQ 3.0 Item 76 and RBS-R item 4 contain jumping and spinning in circles as exemplars). Nevertheless, core autism features (as operationalized by both the SRS total score and multiple RBS-R subscales) demonstrated practically meaningful positive correlations with the majority of sensory constructs considered in the current study. Associated psychiatric symptoms also demonstrated small to moderate correlations with almost all HYPER subconstructs and several modality-specific HYPO and SEEK subconstructs, suggesting that outside of other core autism features, sensory reactivity (particularly HYPER) is most robustly related to transdiagnostic psychiatric symptomatology. Notably, as the sensory subconstructs of Tactile and Movement SEEK demonstrated practically significant positive correlations with externalizing symptoms and features of ADHD but not internalizing symptoms, these two domains may be reflective of an underlying liability for dysregulated or impulsive behavior (see also (80)). These two domains of seeking also showed negative correlations with age, potentially suggesting that these traits decrease over time as children develop increased capacity to regulate their motor impulses with age (e.g., (151,152). Although cross-sectional correlations such as those explored in the current study are insufficient to determine causal relationships between sensory reactivity and other clinical constructs (153), the present findings can nevertheless be useful in generating hypotheses for future targeted investigations of the causal interplay between sensory constructs and other core/associated features of autism.

Despite only two HYPO subconstructs (Speech HYPO and Pain/Temperature HYPO) being considered within the analysis of meta-analytic correlates, it is notable that these two domains of sensory reactivity diverged strongly in terms of their correlations with non-sensory variables. Speech HYPO demonstrated practically significant positive correlations with all core autism features (i.e., SRS, RBS-R subscales) and domains of psychiatric symptoms, as well as practically significant negative correlations with age and VABS socialization. Notably, a child not responding to their name or other speech stimuli is frequently considered a core feature of autism outside of the sensory domain, conceptualized as a failure to orient attention to socially salient stimuli (e.g., (154-156). Thus, it is notable that observed hyporeactivity to speech could feasibly be present in the absence of underlying differences in sensory reactivity (e.g., (due to differences in broader social or attentional processes). Future studies, particularly those that include multi-method assessments of both social-communicative and sensory factors, may be necessary to determine whether the underlying causes of Speech HYPO are indeed sensory in nature, thereby investigating the appropriateness of classifying this subconstruct as a sensory reactivity difference.

Unlike the Speech HYPO domain, the Pain/Temperature HYPO subconstruct was reported at moderately higher levels in autistic children with a categorical label of intellectual disability, and this trait was also significantly negatively associated with all domains of the VABS, though no correlations with core autism features nor psychiatric symptoms reached the threshold for practical significance. Though these results seemingly indicate that insensitivity to pain and temperature covary with intellectual disability and reduced adaptive functioning in the autistic population, we strongly caution against overinterpretation of these findings due to the substantial limitations of quantifying response to pain in autism based on solely reports from caregivers (157, 158). Although a co-occurring diagnosis of intellectual disability or more significant impairments in adaptive behavior may be more common in individuals with additional rare neurological conditions that truly include insensitivity to pain as a symptom (e.g., (congenital insensitivity to pain with anhidrosis (159)), it is also quite possible that proxy reporters such as caregivers underestimate the pain or discomfort of autistic children who are not able to communicate their internal states in typical ways (158). With the recent development of methods to better capture the internal pain experiences of autistic individuals with intellectual disability and/or limited language (160), additional work is greatly needed to determine whether caregiver reports of seeming insensitivity to pain correspond with self-reports of pain experience in this population, providing more conclusive evidence for or against the claim that autistic individuals with more significant cognitive impairments are truly less sensitive to pain and temperature than autistic individuals who are more cognitively-able (versus this difference being driven by atypical *communication* of pain or distress).

Overall, the findings of the current study with regard to studied HYPO subconstructs suggest that Pain/Temperature HYPO and Speech HYPO represent theoretically distinct aspects of the autism phenotype with almost completely non-overlapping correlates and divergent future directions relevant to construct validation. Therefore, for applied researchers hoping to investigate these aspects of the autism phenotype using caregiver-report questionnaires, we strongly recommend that these two HYPO subconstructs in particular be studied at the single-modality level, as the nuanced associations between modality-specific variables and external correlates may be obscured by the use of supra-modal HYPO scores that combine subconstructs into a single variable when assessing individual differences. Notably, it is currently unclear whether these HYPO subconstructs demonstrate equally divergent patterns of external correlations when measured using other techniques (e.g., (clinician observation (83, 84)), and this remains an important avenue for future research.

## Strengths And Limitations

The current study had a number of strengths, including its very large sample size, representation of autistic children and adolescents across a wide range of ages and developmental levels, sensory phenotyping of all major modalities and response patterns with widely used caregiver-report measures, and state-of-the-art statistical approaches that allowed for the pooling of partially overlapping sensory item scores and evaluation of between-dataset effect heterogeneity. However, it was not without limitations. Most notably, the studies that comprised the dataset utilized vastly different methods; each had substantially different inclusion/exclusion criteria, geographic locations, and assessment batteries. To allow for maximal pooling of similar data across studies, we combined measures of the same construct (e.g., (different versions of the same questionnaire, standard scores on different measures of an ostensibly similar construct such as FSIQ or internalizing symptoms into single variables, potentially introducing additional heterogeneity due to noninvariance between the different measures or measure versions. For sensory constructs, this pooling was also done at the item level to allow for different versions of the same measure (i.e., SP1 and SP2, SEQ-2.1 and SEQ-3.0) to be calibrated on the same latent scale using IRT. Though many items on different questionnaire versions were extremely similar, version-specific changes in anchor wording, item stems, or order effects could theoretically have resulted in noninvariance of the two homologous items, again increasing overall heterogeneity. Nevertheless, the random-effects IDA model utilized in the current study allowed for the heterogeneity of each effect to be quantified (i.e., using ICCs and prediction intervals), thereby helping to contextualize both the population summary effect and the range of possible effects observable under different study conditions. The measurement of many sensory subconstructs was also a limitation, as despite the large initial item pool, a number of subconstructs had relatively few initial indicators and were, therefore, difficult to form into viable unidimensional scales from the start. For constructs that could not be operationalized in the current study using a psychometrically adequate unidimensional scale, we opted to use an ad-hoc single item indicator such that these subconstructs would still remain in each supra-modal bifactor model. However, it is unclear whether the use of single-item indicators partially contributed to the psychometric inadequacy of the higher-order HYPO and SEEK constructs, and future studies in which all modality-specific subconstructs are adequately captured are necessary to rule out poor subconstruct measurement as a potential cause of supra-modal construct invalidity for sensory response pattern scores.

Another major limitation of the current study was the fact that multisensory items were removed from the questionnaires before psychometric analyses were undertaken. Although this choice greatly simplified the bifactor models due to the lack of specific-factor cross-loadings, it is notable that “real-world” sensory experiences are inherently multisensory in nature (161). By removing items containing multiple sensory modalities, we may have inadvertently excluded a number of relevant sensory behaviors in real-world contexts from the measurement models, limiting the content validity of the supra-modal constructs operationalized by the general HYPER, HYPO, and SEEK factors. Though it remains unknown whether these items would have been retained in our models based on psychometric criteria or excluded due to misfit, future studies are warranted to investigate the utility and properties of sensory reactivity bifactor (or indeed more complex hierarchically-structured) models that include multisensory items in addition to single-modality subconstructs.

As an additional limitation, the questionnaires used in the current study were all based on caregiver-report of a child’s behavior; even in cases where autistic individuals were capable of self-reporting on their own sensory experiences (or provided such data), this information was not included in the current investigation. As sensory percepts are fundamentally subjective experiences, reports solely based on the observations of untrained proxy reporters (i.e., caregivers) may be capturing only the most extreme and/or distressing sensory reactivity differences, potentially also introducing confounding according to the child’s language or communication ability (see also (162)). Moreover, it is quite possible that our conclusions regarding the inadequacy of supra-modal HYPO and SEEK scores (and/or the adequacy of supra-modal HYPER scores) are limited to caregiver-reported sensory measures, and additional work is needed to test the appropriateness of such scores using other measurement methods, including self-report (e.g., (163, 164), clinician-rated behavioral observation (e.g., (83,84,165,166), and parent/caregiver interview (e.g., (83) tools. Ideally, further studies of autistic youth and adults capable of self-report should attempt to utilize multimodal sensory measurements that include both self- *and* informant-reports simultaneously ((e.g., (167) see also (83) for a measure combining clinician observation with caregiver interview), therefore allowing both an individual’s internal experience and observed behavior to contribute to their ratings of sensory reactivity.

Considering the statistical limitations of the study, it is worth noting that all associations between caregiver-reported sensory reactivity differences and clinical/demographic variables were estimated using models that did not control for other relevant demographic or clinical variables (e.g., (age, sex, IQ/DQ, or language level). Thus, many of the meta-analytic correlation estimates in our current study likely overestimate the strength of hypothesized causal effects (i.e., associations in which the sensory construct is theorized to be causally upstream of the correlate of interest (168)) due to the presence of (often substantial) residual confounding (153). On the other hand, the current study only examined unconditional, linear associations between variables; therefore, it is also possible that the strength of any *nonlinear* relationship was underestimated. Future work should attempt to quantify the conditional linear and nonlinear effects of various sensory predictors on relevant clinical outcomes over and above other potentially confounding variables (e.g., (102). Lastly, it is notable that the current investigation relied solely on cross-sectional data, limiting our ability to draw conclusions regarding the predictive validity of sensory reactivity for other relevant outcomes. Although some studies have begun to demonstrate the predictive utility of sensory reactivity in autistic children and other populations such as infants at elevated likelihood to develop autism (e.g., (169-174), these studies have largely used supra-modal response pattern scores; therefore, additional large-scale, longitudinal studies are necessary to determine which single-modality sensory subconstructs (or combinations thereof) can be utilized as clinically-relevant predictors of core and frequently co-occurring features of autism.

## Conclusion

The past decade has seen a substantial rise in the number of studies examining the sensory aspects of autism (28), but to date, relatively little published work has examined the latent structure or construct validity of proposed sensory (sub)constructs, particularly those that span multiple sensory modalities. By compiling a large dataset of richly-phenotyped autistic children, we conducted an integrative data analysis that specifically investigated the hierarchical structure of the three canonical sensory “response patterns” (i.e., HYPER, HYPO, and SEEK). Although much research to date has focused on the examination of response patterns that span multiple modalities, the current study demonstrates that some of these *supra-modal construct scores* (in particular those purported to tap hyporeactivity and to a lesser extent, sensory seeking) are contaminated to a substantial degree by modality-specific variance, making these supra-modal scores difficult to interpret when such variance is not explicitly partialed out (e.g., (in the context of a latent variable model). Depending upon the nature of the research question (e.g., (if assessing sensory correlates within the same modality or a mechanism of change that is likely to work at the level of a single modality rather than the supra-modal level), *modality-specific subconstruct scores* may be preferable, or at least represent a viable alternative to supra-modal scores for characterizing individual differences in sensory reactivity in autistic children and adolescents, although additional research is needed to further develop modality-specific sensory measures beyond the limited subsets of items available in broadband inventories currently in use. We therefore recommend that applied researchers studying the sensory aspects of autism tailor the sensory reactivity construct(s) they hope to measure to their specific research questions, rather than exclusively and uncritically relying on supra-modal response pattern scores.

Using integrative data analysis models, we also examined meta-analytic bivariate associations between single-modality sensory subconstructs and various other clinical outcomes, with other measures of core autism features (e.g., (subscales of the RBS-R) and psychiatric symptoms demonstrating particularly strong relations with most aspects of sensory reactivity. Although the empirically derived sensory subconstruct measures in the current study correlated meaningfully with other clinical outcomes, there remains a great need to expand existing measures and/or develop novel measures that sample each modality-specific subconstruct (and when relevant, multiple distinct aspects or subdimensions of that subconstruct) in greater detail, as well as to explicitly investigate caregiver-reported reactivity in multisensory contexts that were not tested in the current study. Notably, the field of sensory research remains ripe for cross-disciplinary collaboration between clinical and behavioral scientists, occupational therapists, psychologists, neuroscientists, and autistic individuals themselves (e.g., (175-179), as a synthesis of clinical, behavioral, neuroscientific, and lived experience perspectives on sensory reactivity within and across modalities is likely to produce valid and useful assessments of specific aspects of the autism phenotype, their underlying psychological and neural mechanisms, and their unique clinical correlates. Basic and applied research into the sensory features of autism has immense potential to improve the lives of many autistic individuals across the lifespan, but in order to realize this potential, systematic efforts must be made to rigorously define all sensory constructs of interest and develop psychometrically valid measures of such constructs for use in both research and clinical practice.

## Figures and Tables

**Table 1:** Participant Demographics and Broader Characteristics for Calibration Sample and Full Sample

	Calibration Sample	Full Sample
Sample Size	1197	3866
Age (years; *M* ± *SD* (*n*), min–max)	8.36 ± 3.29 (1197), 3.00–17.97	8.41 ± 3.36 (3866), 3.00–17.99
Sex (male / female; *n* [%])	870 (72.7%) / 326 (27.3%)	3072 (79.5%) / 793 (20.5%)
**Ethnicity**		
Hispanic or Latino (*n* [%])	53 (4.4%)	284 (7.3%)
Not Hispanic or Latino (*n* [%])	575 (48.0%)	1957 (50.6%)
Not Reported or Unknown (*n* [%])	569 (47.5%)	1625 (42.0%)
**Race**		
White (*n* [%])	783 (65.4%)	2919 (75.5%)
American Indian or Alaska Native (*n* [%])	7 (0.6%)	41 (1.1%)
Asian (*n* [%])	33 (2.8%)	106 (2.7%)
Black or African American (*n* [%])	41 (3.4%)	109 (2.8%)
Native Hawaiian or Other Pacific Islander (*n* [%])	2 (0.2%)	4 (0.1%)
More than One Race (*n* [%])	100 (8.4%)	279 (7.2%)
Not Reported or Unknown (*n* [%])	231 (19.3%)	408 (10.6%)
**Sensory Measures Administered**		
Sensory Profile 1/ Short Sensory Profile 1 (*n* [%])	905 (75.6%)	1573 (40.0%)
Sensory Profile 2/ Short Sensory Profile 2 (*n* [%])	293 (24.5%)	293 (7.6%)
Sensory Experiences Questionnaire Version 2.1 (*n* [%])	420 (35.1%)	433 (11.2%)
Sensory Experiences Questionnaire Version 3.0 (*n* [%])	510 (42.6%)	2498 (64.6%)
**Cognitive Scores**		
Intelligence Quotient (IQ; *n* [%])	503 (42.0%)	1042 (27.0%)
Developmental Quotient (DQ; *n* [%])	125 (10.4%)	247 (6.4%)
Neither (*n* [%])	569 (47.5%)	2577 (66.7%)
Full-scale IQ/DQ (*M* ± *SD* (*n*), min–max)	91.3 ± 25.8 (414), 12.0-153.0	92.1 ± 24.5 (1028), 12.0-153.0
Verbal IQ/DQ (*M* ± *SD* (*n*), min–max)	83.9 ± 30.6 (451), 8.0-153.0	88.8 ± 27.9 (1038), 8.0-160.0
Nonverbal IQ/DQ (*M* ± *SD* (*n*), min–max)	92.4 ± 25.5 (600), 16.7–148.0	93.0 ± 26.0 (1193), 6.0-160.0
**Adaptive Functioning**		
Vineland Adaptive Behavior Composite (*M* ± *SD* (*n*), min–max)	71.0 ± 16.6 (367), 22.0-121.0	69.7 ± 16.1 (1472), 20.0-126.0
Vineland Communication (*M* ± *SD* (*n*), min–max)	76.6 ± 17.5 (472), 22.0-133.0	73.3 ± 19.6 (1590), 20.0-133.0
Vineland Daily Living Skills (*M* ± *SD* (*n*), min–max)	74.6 ± 19.2 (339), 19.0-125.0	72.3 ± 19.1 (1449), 19.0-138.0
Vineland Socialization (*M* ± *SD* (*n*), min–max)	71.3 ± 15.5 (471), 32.0-127.0	68.8 ± 17.1 (1589), 20.0-127.0
**Psychiatric Symptoms**		
Total Psychiatric Symptoms (T-score; *M* ± *SD* (*n*), min–max)	64.1 ± 11.4 (298), 31.0-111.0	63.6 ± 10.4 (671), 26.0-111.0
Internalizing Symptoms (T-score; *M* ± *SD* (*n*), min–max)	59.3 ± 10.7 (392), 33.0–98.0	59.9 ± 11.9 (765), 0.0-106.0
Externalizing Symptoms (T-score; *M* ± *SD* (*n*), min–max)	57.3 ± 11.5 (393), 32.0-112.0	56.5 ± 12.2 (766), 0.0-112.0

*Note*. The calibration sample was used to perform the empirically-driven item reduction for each subconstruct scale, whereas the full sample was used to examine correlates of each sensory construct. Cognitive scores (varied standardized measures of both IQ and DQ; see methods section for additional details) and adaptive behavior scores (derived from the Vineland Adaptive Behavior Scales; Sparrow, 2011) are presented on a standard score metric (normative sample *M* = 100, *SD* = 15). Psychiatric symptom scores (varied standardized measures; see methods section for additional details) are presented on a T-score metric (normative sample *M* = 50, *SD* = 10). Full demographics for each subsample can be found in Supplemental Table S1.

**Table 2:** Results of Scale Refinement Process for Each Single-modality Sensory Subconstruct

	Initial *n*_items_	Final *n*_items_	Final Model Fit			ρ_xx_ / ω_⊤_	Scale Retained?
HYPER Constructs			TLI_C2_	RMSEA_C2_	SRMR			
Auditory (Bifactor)	10	7	0.989	0.051	0.046	0.893 / 0.945	YES
Unidimensional Model	10	4	0.998	0.030	0.028	0.875 / 0.898	YES^a^
Visual	9	4	0.996	0.046	0.021	0.877 / 0.928	YES
Tactile (Bifactor)	18	13	0.985	0.043	0.047	0.916 / 0.946	YES
Unidimensional Model	18	4	0.978	0.053	0.038	0.718 / 0.713	YES^a^
Olfactory	3	3	—	—	0.017	0.751 / 0.840	YES
Gustatory	9	4	1.005	0.000	0.017	0.849 / 0.859	YES
Vestibular/Proprioceptive [Movement]	10	4	1.002	0.000	0.030	0.757 / 0.891	YES
**HYPO Constructs**							
Auditory	5	3	—	—	0.024	0.891 / 0.904	YES (Speech)
Visual	3	3	—	—	0.024	**0.656 / 0.631**	**NO**
Tactile/Somatosensory	9	3	—	—	0.022	0.837 / 0.863	YES (Pain/Temperature)
Olfactory	2	**2**	—	—	—	—	**NO**
Gustatory	1	**1**	—	—	—	—	**NO**
**SEEK Constructs**							
Auditory	3	3	—	—	0.010	0.729 / **0.623**	**NO**
Visual	12	6	1.002	0.000	0.027	0.834 / 0.859	YES
Tactile	10	4	1.011	0.000	0.039	0.746 / 0.727	YES
Olfactory	5	3	—	—	**0.045**	—	**NO**
Gustatory	5	3	—	—	**0.044**	—	**NO**
Oral Tactile	5	4	0.985	0.088	0.026	0.848 / 0.938	YES
Vestibular/Proprioceptive [Movement]	10	4	0.993	0.042	0.029	0.831 / 0.818	YES

*Note*. HYPER = hyperreactivity; HYPO = hyporeactivity; SEEK = sensory seeking. Bolded values indicate instances wherein a scale did not meet a priori-specified criteria for psychometric adequacy (i.e., TLI_C2_ > 0.97, RMSEA_C2_ < 0.089, SRMR < 0.05 [or < 0.033 for 3-item composites], ρ_xx_ > 0.7, and ω_⊤_ > 0.7). *n*_items_ = number of items on the scale; TLI_C2_ = *C*_2_-statistic based Tucker-Lewis index; TLI_C2_ = (*C*_2_-statistic based root mean square error of approximation; SRMR = limited-information standardized root-mean-square residual; ρ_xx_ = marginal reliability; ω_⊤_ = omega total (composite score reliability).

a As a bifactor model of these constructs demonstrated adequate fit and stronger reliability, unidimensional Auditory and Tactile HYPER scores were retained for use in bifactor HYPER model only.

**Table 3:** Factor Loadings and Bifactor Coefficients for Hyperreactivity (HYPER) Items

Item	G- HYPER	Auditory	Visual	Tactile	Gustatory	Olfactory	Movement	*h* ^2^	*I-ECV*
SP1 Q1/SP2 Q1	0.513	0.699	—	—	—	—	—	0.752	0.350
SP1 Q2/SP2 Q2	0.469	0.683	—	—	—	—	—	0.687	0.320
SEQ2 Q1/SEQ3 Q1	0.469	0.753	—	—	—	—	—	0.786	0.279
SEQ3 Q9	0.611	0.475	—	—	—	—	—	0.599	0.622
SEQ2 Q8/SEQ3 Q15	0.571	—	0.594	—	—	—	—	0.679	0.480
SP1 Q10/SP2 Q15	0.563	—	0.677	—	—	—	—	0.775	0.409
SP1 Q14/SP2 Q13	0.649	—	0.601	—	—	—	—	0.783	0.538
SP1 Q15	0.565	—	0.599	—	—	—	—	0.677	0.471
SP1 Q30/SP2 Q16/SEQ2 Q15/SEQ3 Q49	0.449	—	—	0.449	—	—	—	0.404	0.500
SP1 Q36/SP2 Q18	0.608	—	—	0.261	—	—	—	0.438	0.844
SEQ2 Q16/SEQ3 Q38	0.564	—	—	0.100	—	—	—	0.328	0.970
SP1 Q33	0.650	—	—	0.324	—	—	—	0.527	0.801
SEQ2 Q22/SEQ3 Q59	0.451	—	—	—	0.695	—	—	0.686	0.297
SP1 Q55/SP2 Q44	0.465	—	—	—	0.786	—	—	0.834	0.259
SP1 Q56/SP2 Q45	0.454	—	—	—	0.721	—	—	0.727	0.284
SEQ3 Q70	0.482	—	—	—	0.393	—	—	0.387	0.600
SEQ3 Q61	0.619	—	—	—	—	0.685	—	0.851	0.450
SEQ3 Q66	0.543	—	—	—	—	0.713	—	0.803	0.367
SEQ3 Q73	0.547	—	—	—	—	0.413	—	0.469	0.637
SP1 Q18	0.482	—	—	—	—	—	0.701	0.724	0.321
SP1 Q19	0.462	—	—	—	—	—	0.680	0.677	0.316
SP1 Q20	0.408	—	—	—	—	—	0.637	0.572	0.291
SEQ3 Q83	0.464	—	—	—	—	—	0.621	0.600	0.359
**Bifactor Coefficients**									
ω_⊤_/ω_S_	0.986	0.911	0.938	0.743	0.908	0.851	0.906		
ω_H_/ω_HS_	0.800	0.552	0.470	0.142	0.596	0.443	0.601		
*ECV*_G_	0.436	0.381	0.475	0.773	0.326	0.460	0.322		
*ECV*_SS_	—	0.619	0.525	0.227	0.674	0.540	0.678		

*Note*. Factor loadings are derived from full-information maximum likelihood confirmatory bifactor analysis, equivalent to a bifactor graded response model. SP = Sensory Profile; SEQ = Sensory Experiences Questionnaire; *h*^2^ = communality; *I-ECV =* item explained common variance; G-HYPER = General HYPER domain factor; ω_⊤_ = Coefficient omega total (total score reliability); ω_S_ = Coefficient omega subscale (subscale reliability); ω_H_ = Coefficient omega hierarchical (general factor saturation; ≥ 0.8 indicates strong general factor); ω_HS_ = Coefficient omega hierarchical subscale (specific factor saturation; ≥ 0.20/0.25 support the added value of a specific factor under conditions of high/low reliability, respectively); *ECV*_G_ = General factor explained common variance; *ECV*_SS_ = Specific factor explained common variance for a subscale (≥ 0.30/0.45 support the added value of a specific factor under conditions of high/low reliability, respectively).

**Table 4:** Factor Loadings and Bifactor Coefficients for Hyporeactivity (HYPO) Items

Item	G-HYPO	Speech	Pain/Temp	*h^2^*	*I-ECV*
SEQ2 Q3/SEQ3 Q8	0.446	0.725	—	0.724	0.275
SP1 Q6/SP2 Q6	0.344	0.726	—	0.646	0.183
SP1 Q7/SP2 Q7	0.416	0.848	—	0.892	0.194
SEQ2 Q19/SEQ3 Q53	0.413	—	0.690	0.647	0.264
SP1 Q42/SP2 Q23/SP2 Q24	0.441	—	0.870	0.951	0.205
SEQ3 Q56	0.447	—	0.478	0.428	0.466
SEQ2 Q10/SEQ3 Q22/SEQ3 Q23 [Visual]	0.541	—	—	0.292	1.000
SP1 Q46/SP2 Q26 [Tactile]	0.470	—	—	0.221	1.000
SEQ2 Q4/SEQ3 Q4 [Auditory]	0.554	—	—	0.307	1.000
SEQ3 Q69 [Olfactory]	0.445	—	—	0.198	1.000
SEQ3 Q74 [Gustatory]	0.448	—	—	0.201	1.000
**Bifactor Coefficients**					
ω_⊤_/ω_S_	0.905	0.929	0.845		
ω_H_/ω_HS_	0.611	0.710	0.579		
*ECV*_G_	0.413	0.219	0.285		
*ECV*_SS_	—	0.781	0.715		

*Note*. Factor loadings are derived from full-information maximum likelihood confirmatory bifactor analysis, equivalent to a bifactor graded response model. SP = Sensory Profile; SEQ = Sensory Experiences Questionnaire; *h*^2^ = communality; *I-ECV =* item explained common variance; G-HYPO = General HYPO domain factor; ω_⊤_ = Coefficient omega total (total score reliability); ω_S_ = Coefficient omega subscale (subscale reliability); ω_H_ = Coefficient omega hierarchical (general factor saturation; ≥ 0.8 indicates strong general factor); ω_HS_ = Coefficient omega hierarchical subscale (specific factor saturation; ≥ 0.20/0.25 support the added value of a specific factor under conditions of high/low reliability, respectively); *ECV*_G_ = General factor explained common variance; *ECV*_SS_ = Specific factor explained common variance for a subscale (≥ 0.30/0.45 support the added value of a specific factor under conditions of high/low reliability, respectively).

**Table 5:** Factor Loadings and Bifactor Coefficients for Sensory Seeking (SEEK) Items (Excluding Single-item Indicators

Item	G- SEEK	Visual	Tactile	Oral Tactile	Movement	*h^2^*	*I-ECV*
SEQ2 Q9/SEQ3 Q17	0.691	0.409	—	—	—	0.645	0.740
SP1 Q97/SP2 Q80/SP2 Q81	0.593	0.296	—	—	—	0.439	0.801
SEQ3 Q19	0.656	0.310	—	—	—	0.527	0.817
SEQ3 Q27	0.552	0.558	—	—	—	0.616	0.494
SEQ3 Q29	0.526	0.487	—	—	—	0.513	0.538
SEQ3 Q30	0.572	0.486	—	—	—	0.563	0.581
SP1 Q45/SP1 Q45/SP2 Q21/SP2 Q25	0.598	—	0.364	—	—	0.490	0.729
SEQ2 Q36f/SEQ3 Q45	0.551	—	0.688	—	—	0.777	0.390
SEQ3 Q37	0.436	—	0.413	—	—	0.360	0.527
SEQ3 Q50	0.672	—	0.131	—	—	0.469	0.963
SEQ2 Q25/SEQ3 Q62	0.441	—	—	0.796	—	0.827	0.235
SP1 Q64	0.433	—	—	0.856	—	0.920	0.203
SP1 Q65	0.421	—	—	0.772	—	0.774	0.229
SEQ3 Q71	0.525	—	—	0.706	—	0.774	0.356
SP1 Q24/SP1 Q25/SP2 Q27	0.611	—	—	—	0.483	0.606	0.615
SEQ2 Q27/SEQ3 Q76	0.624	—	—	—	0.358	0.518	0.752
SP1 Q84/SP2 Q60	0.599	—	—	—	0.160	0.385	0.933
SP1 Q26	0.683	—	—	—	0.651	0.890	0.524
**Bifactor Coefficients**							
ω_⊤_/ω_S_	0.969	0.876	0.751	0.934	0.840		
ω_H_/ω_HS_	0.799	0.287	0.255	0.686	0.241		
*ECV*_G_	0.531	0.656	0.620	0.253	0.663		
*ECV*_SS_	—	0.344	0.380	0.747	0.337	

*Note*. Factor loadings are derived from full-information maximum likelihood confirmatory bifactor analysis, equivalent to a bifactor graded response model. SP = Sensory Profile; SEQ = Sensory Experiences Questionnaire; *h*^2^ = communality; *I-ECV =* item explained common variance; G-SEEK = General SEEK domain factor; ω_⊤_ = Coefficient omega total (total score reliability); ω_S_ = Coefficient omega subscale (subscale reliability); ω_H_ = Coefficient omega hierarchical (general factor saturation; ≥ 0.8 indicates strong general factor); ω_HS_ = Coefficient omega hierarchical subscale (specific factor saturation; ≥ 0.20/0.25 support the added value of a specific factor under conditions of high/low reliability, respectively); *ECV*_G_ = General factor explained common variance; *ECV*_SS_ = Specific factor explained common variance for a subscale (≥ 0.30/0.45 support the added value of a specific factor under conditions of high/low reliability, respectively).

## Abbreviations

ABC: Adaptive Behavior Composite
ADHD: Attention-Deficit/Hyperactivity Disorder
CFA: Confirmatory Factor Analysis
COM: Communication
CFI: Comparative Fit Index
CrI: Credible Interval
DLS: Daily Living Skills
EAP: Expected A Posteriori
ECV: Explained Common Variance
GRM: Graded Response Model
HYPER: Hyperreactivity
HYPER: Hyporeactivity
FSDQ: Full-scale Developmental Quotient
FSIQ: Full-scale Intelligence Quotient
IDA: Integrative Data Analysis
INT: Internalizing Symptoms
IRT: Item Response Theory
NVDQ: Nonverbal Developmental Quotient
NVIQ: Nonverbal Intelligence Quotient
ROPE: Region of Practical Equivalence [to zero]
RBS-R: Repetitive Behavior Scale–Revised
MLR: Robust Maximum Likelihood
RMSEA: Root Mean Square Error of Approximation
*BF*_ROPE_: ROPE Bayes Factor
RSM: Repetitive Sensorimotor Behavior
RSC: Ritutalistic/Sameness/Compulsive Behavior
SIB: Self-injurious Behavior
SEQ-2.1: Sensory Experiences Questionnaire, version 2.1
SEQ-3.0: Sensory Experiences Questionnaire, version 3.0
[S]SP1: [Short] Sensory Profile 1
[S]SP2: [Short] Sensory Profile 2
SP-3D:I: Sensory Processing–Three Dimensions Scale: Inventory
SEEK: Sensory Seeking
SOC: Socialization
SRS: Social Responsiveness Scale
SRMR: Standardized Root Mean Square Residual
TLI: Tucker-Lewis Index
VDQ: Verbal Developmental Quotient
VIQ: Verbal Intelligence Quotient
VABS: Vineland Adaptive Behavior Scales
